# Ferroptosis–immune crosstalk in ionizing radiation–induced testicular injury: mechanisms and therapeutic opportunities

**DOI:** 10.3389/fimmu.2026.1935866

**Published:** 2026-09-22

**Authors:** Rong Xu, Changhao He, Shuhang Feng, Yixuan Cai, Qiming Zhao, Gaojie Xin, Quanlong Zhang, Luping Qin, Lei Wang

**Affiliations:** 1 School of Pharmaceutical Sciences, Zhejiang Chinese Medical University, Hangzhou, China; 2 Zhejiang Key Laboratory of Chinese Medicine Modernization, School of Pharmaceutical Sciences, Zhejiang Chinese Medical University, Hangzhou, China

**Keywords:** blood–testis barrier, ferroptosis, immune crosstalk, ionizing radiation, macrophages, radioprotection, sertoli cells, testicular injury

## Abstract

Accumulating evidence suggests that ferroptosis—an iron–dependent, lipid peroxidation–driven form of regulated cell death—may contribute substantially to ionizing radiation (IR)–induced testicular injury. In this review, we develop a hypothesis–generating framework for ferroptosis–immune crosstalk in the testis. Within this framework, ferroptotic injury to Sertoli cells is proposed to compromise the blood–testis barrier (BTB), potentially facilitating autoantigen exposure and adaptive immune activation; reciprocal inflammatory signaling may, in turn, increase the susceptibility of neighboring cells to ferroptosis. Potential interactions between ferroptosis and apoptosis, autophagy, pyroptosis, and necroptosis are considered as additional components of this proposed network. We summarize clinically used drugs and emerging strategies targeting ferroptosis, including iron chelators, radical scavengers, NRF2 activators, natural antioxidants, and nanomedicine. Key challenges such as the temporal dynamics of ferroptosis, dose–dependent effects, and translational gaps are discussed. This framework is intended to organize current evidence, identify testable mechanistic links, and guide future studies of male fertility preservation through ferroptosis–immune modulation.

## 1 Introduction

Over the past five decades, male reproductive health has faced severe challenges worldwide, with declining semen quality and decreasing fertility rates emerging as critical issues in international public health (1, 2). Epidemiological studies indicate that approximately 7% to 12% of men of reproductive age worldwide are affected by infertility, and male factors account for about 50% of infertility cases among infertile couples (3, 4). These trends highlight that declining male reproductive capacity represents a global public health concern rather than a region–specific issue. The increasing reliance on medical radiation, occupational exposure in radiation–related industries, and emerging concerns regarding long–term radiation safety have further emphasized the need to understand and prevent radiation–associated reproductive damage.

Among various physicochemical environmental factors, ionizing radiation (IR) is increasingly recognized as a major risk to male reproductive safety, owing to its extensive applications in radiodiagnosis and radiotherapy, the nuclear industry, and aerospace (5). With the increasing use of radiotherapy in cancer treatment and the expanding population exposed to occupational or environmental radiation, protection of reproductive function has become an important component of radiation health management. However, current radioprotective strategies mainly focus on acute tissue toxicity and genomic damage, whereas long–term consequences on male reproductive capacity remain insufficiently understood. The testis is one of the most radiation–sensitive organs in the human body, with its highly proliferative spermatogenic epithelium being susceptible to pronounced oxidative stress, DNA damage, and spermatogenic impairment even at low doses, leading to reduced sperm concentration, increased morphological abnormalities, and even azoospermia, thereby posing a serious threat to male reproductive health (6, 7). Conventional views have emphasized apoptosis as a major mechanism of IR–induced germ cell loss; however, increasing evidence indicates that oxidative stress, inflammatory activation, and disruption of tissue homeostasis also contribute to persistent reproductive dysfunction after irradiation.

Although apoptosis has been considered a major mechanism responsible for radiation–induced germ cell loss, accumulating evidence indicates that IR–induced reproductive toxicity involves more complex and persistent biological responses, including oxidative stress amplification, inflammatory activation, mitochondrial dysfunction, and disruption of tissue–specific homeostasis (5, 8, 9). Recent studies in different organs have highlighted that ferroptosis represents an important contributor to radiation–associated tissue injury, particularly through iron dysregulation, lipid peroxidation, and impairment of antioxidant defense systems (10). However, current knowledge regarding ferroptosis in radiation–induced male reproductive injury remains limited, and whether ferroptosis interacts with the unique immune–privileged environment of the testis has not been systematically explored (11).

In recent years, the discovery of ferroptosis—a novel form of regulated cell death driven by iron-dependent lipid peroxidation—has provided a revolutionary new perspective for understanding the mechanisms of radiation-induced injury (12). Evidence from radiation biology and ferroptosis research supports several mechanistic links that may converge in the irradiated testis. IR can directly generate hydroxyl radicals (·OH) through water radiolysis, while radiation–associated DNA damage has been linked to suppression of the System Xc^-^/SLC7A11–GSH–GPX4 antioxidant axis and perturbation of intracellular iron homeostasis. In parallel, NCOA4–mediated ferritinophagy provides a potential route for increasing the labile iron pool and promoting Fenton chemistry. Given the enrichment of polyunsaturated fatty acids (PUFAs), including docosahexaenoic acid (DHA), and the high dependence of spermatogenesis on iron metabolism, these observations support—but do not yet establish—the possibility that these pathways converge to enhance ferroptotic susceptibility in testicular cells, particularly Sertoli cells (13, 14).

However, ferroptosis is by no means an isolated cellular metabolic event; its deep interaction with the immune system is progressively being elucidated (15). Owing to its distinctive mode of plasma membrane rupture, ferroptosis releases copious damage-associated molecular patterns (DAMPs, such as HMGB1 and mtDNA) that possess intrinsic pro-inflammatory immunogenicity. In the testis, which harbors a highly specialized immune–privileged microenvironment, these observations raise the possibility of a particularly important interaction between ferroptotic injury and immune dysregulation. Under physiological conditions, the BTB and the local immunoregulatory environment restrict inappropriate immune responses against germ–cell antigens (16). We propose that, following IR exposure, ferroptotic injury to Sertoli cells may compromise BTB integrity, whereas DAMPs released from injured or ferroptotic cells may favor pro–inflammatory activation of resident macrophages. Cytokines and reactive species generated within this inflammatory microenvironment could, in turn, weaken antioxidant defenses such as the GPX4 system in neighboring cells and thereby increase their susceptibility to secondary ferroptosis (17). Together, these observations form the basis of a proposed positive–feedback model linking ferroptosis, immune activation, BTB dysfunction, and further cellular injury. Importantly, the complete sequence of this proposed loop has not yet been demonstrated longitudinally in the irradiated testis and should therefore be regarded as a testable conceptual framework rather than an established causal pathway.

Although individual components of ferroptosis, oxidative stress, immune activation, and BTB dysfunction have been described in radiation injury or male reproductive disorders, whether these processes operate as an integrated ferroptosis–immune crosstalk network in the irradiated testis remains unresolved. Existing studies are largely fragmented across individual pathways, cell types, or disease models, and direct spatiotemporal evidence linking these events within the testicular microenvironment is still limited.

This review aims to systematically delineate the landscape of ferroptosis–immune crosstalk in IR–induced testicular injury. We first outline the metabolic context and candidate molecular mechanisms that may link radiation exposure to testicular ferroptosis; subsequently, we examine the potential interactions among ferroptosis, DAMP signaling, testicular macrophages, T cells, and Sertoli cells, and consider how these processes may contribute to the loss of immune privilege and BTB dysfunction. By integrating these lines of evidence, we propose a hypothesis–generating framework centered on the ferroptosis–immune axis. This conceptual framework, summarized in **Figure 1**, connects radiation–induced oxidative and iron–dependent stress with cell–specific ferroptotic injury, BTB dysfunction, DAMP–mediated innate immune activation, adaptive immune responses, and secondary cellular injury. Rather than defining an established causal pathway, the framework is intended to organize currently fragmented observations and highlight mechanistic links that require direct validation. Potential therapeutic intervention points within this network are mapped separately in **Figure 2** to distinguish candidate targets from the proposed pathogenic framework itself.

**Figure 1:**
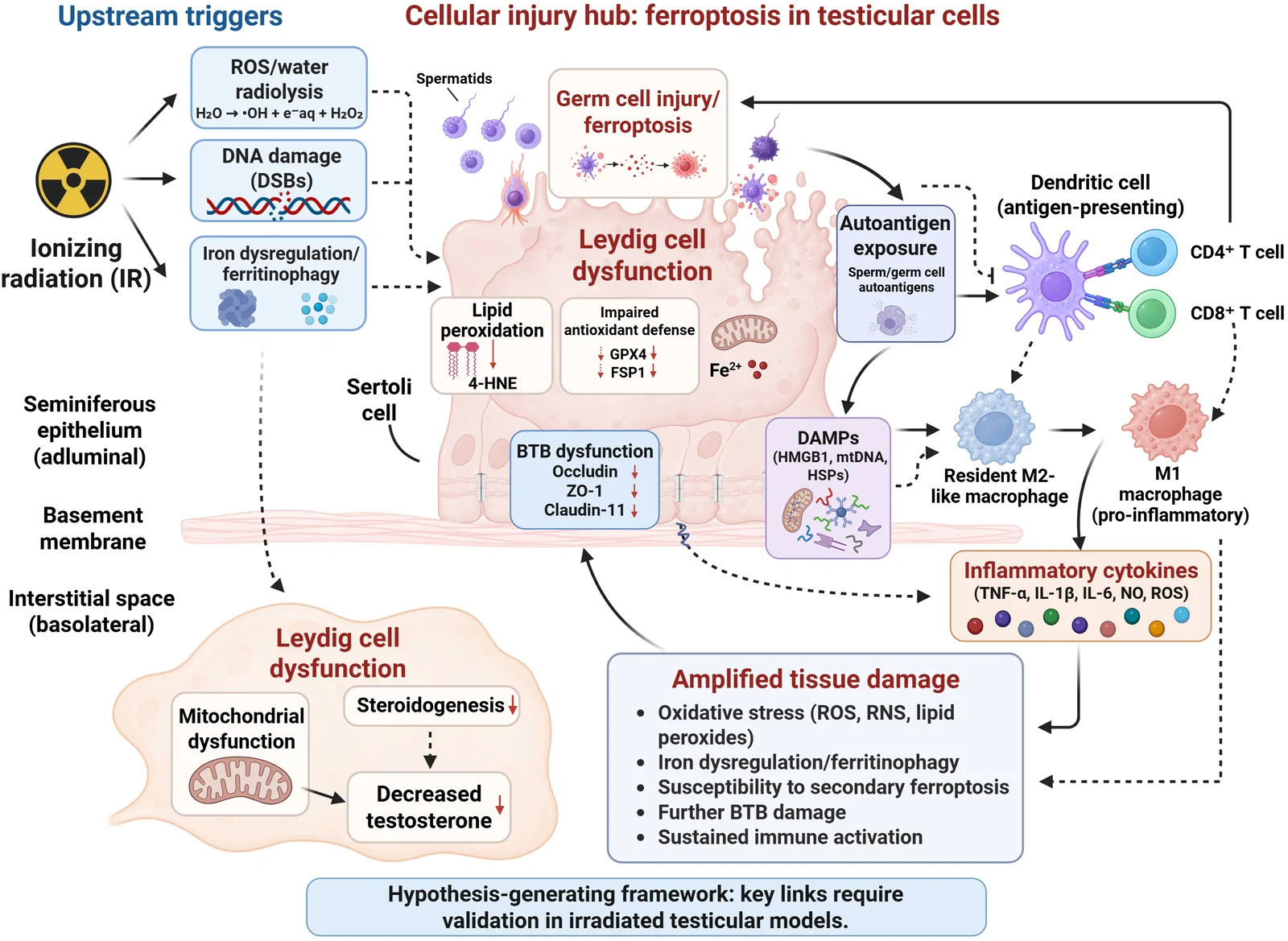
Hypothesis-generating conceptual framework of ferroptosis-immune crosstalk in ionizing radiation-induced testicular injury. In the seminiferous epithelium, IR-induced lipid peroxidation in Sertoli cells generates 4-HNE, which suppresses GPX4 activity and triggers the degradation of tight junction proteins (Occludin, ZO-1, and Claudin-11), leading to the anatomical disruption of the BTB. The loss of BTB integrity allows sperm autoantigens to leak from the adluminal compartment into the interstitial space, where they are captured by dendritic cells and presented to CD4^+^ and CD8^+^ T cells, initiating adaptive immune activation. Concurrently, ferroptosis of germ cells (spermatocytes and spermatids) releases large amounts of damage-associated molecular patterns (DAMPs), including HMGB1, mtDNA, and heat shock proteins (HSPs). In the interstitial space, these DAMPs and inflammatory signals drive the polarization of testicular resident M2-like macrophages into a pro-inflammatory M1 phenotype. The M1 macrophages secrete pro-inflammatory cytokines (TNF-α, IL-6, NO), which further amplify Sertoli cell ferroptosis and BTB damage. Additionally, these inflammatory cytokines impair Leydig cell function by inducing mitochondrial damage and inhibiting steroidogenic acute regulatory protein (StAR) and 3β-hydroxysteroid dehydrogenase (3β-HSD) activity, resulting in decreased testosterone synthesis. Together, these interactions form a proposed positive-feedback model in which ferroptotic injury may promote BTB dysfunction, autoantigen exposure, and inflammatory activation, which could in turn increase susceptibility to further ferroptosis. Conceptual connections in the figure represent mechanistic relationships that remain to be directly validated in irradiated testicular tissue. The diagram is intended as a hypothesis-generating framework rather than an established causal pathway. Solid arrows indicate relationships supported by relatively direct experimental evidence, whereas dashed arrows indicate proposed or indirectly supported connections that require further validation in irradiated testicular models. Some graphical elements were created with BioRender.com.

**Figure 2:**
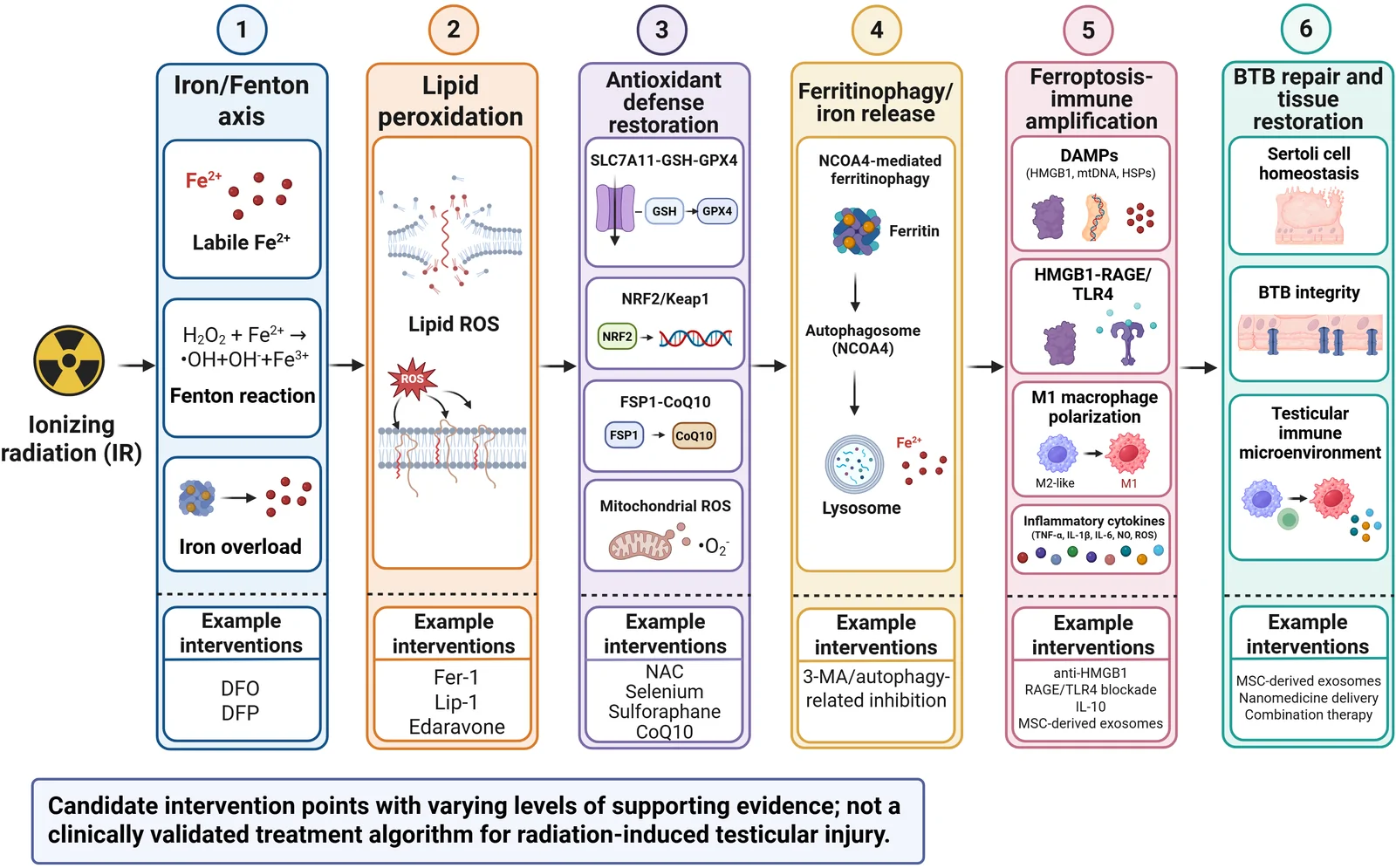
Potential intervention targets within the proposed ferroptosis-immune crosstalk framework. Candidate therapeutic strategies are mapped to distinct pathological nodes of IR-induced testicular injury. Upstream interventions target labile iron accumulation and Fenton chemistry, lipid peroxidation, NCOA4-mediated ferritinophagy, mitochondrial oxidative stress, and impaired SLC7A11-GSH-GPX4/FSP1 antioxidant defenses. Downstream strategies target DAMP-associated HMGB1-RAGE/TLR4 signaling, pro-inflammatory macrophage activation, inflammatory cytokine amplification, and disruption of the blood-testis barrier. Tissue-repair and delivery approaches, including MSC-derived exosomes and targeted nanomedicine, may provide additional strategies for restoring the spermatogenic microenvironment. The diagram summarizes candidate intervention points and does not imply that these approaches have been clinically validated for radiation-induced testicular injury. Some graphical elements were created with BioRender.com.

## 2 Testis–specific immune–privileged microenvironment

As a highly specialized organ in the male reproductive system, the testis not only undertakes key physiological functions such as spermatogenesis and androgen synthesis, but also establishes a significantly tissue–specific microenvironment. This microenvironment is collectively formed by the blood–testis barrier (BTB), local immune tolerance network, and unique metabolic compartmentalization, and its core function is to maintain the stability of spermatogenesis and immune homeostasis (16, 18). At the same time, these highly refined structural and metabolic features also render the testis highly susceptible to pathological stimuli such as IR, oxidative stress, and ferroptosis (19).

### 2.1 Physical and immune isolation mediated by the BTB

The blood–testis barrier is the core structural basis for testicular immune privilege and represents one of the most complex epithelial barriers in mammals. The BTB is primarily composed of tight junctions, adherens junctions, gap junctions, and desmosome–like junctions formed at the basolateral aspect of adjacent Sertoli cells. Its function is not limited to mechanical isolation but also involves local substance exchange, signal transduction, and maintenance of microenvironmental homeostasis (16).

Structurally, the BTB divides the seminiferous epithelium into a basal compartment and an adluminal compartment. Spermatogonia and early primary spermatocytes reside in the basal compartment, whereas post–meiotic haploid germ cells migrate into the adluminal compartment to continue their development. Because haploid spermatids and spermatozoa formed after meiosis express numerous specific antigens that appear only after puberty—antigens not fully recognized during the establishment of immune tolerance—they possess inherent autoimmunogenic potential (20). The BTB effectively prevents immune cells, antibodies, and circulating macromolecules from entering the adluminal compartment, thereby maintaining the “immune privilege” status of the testis (16).

### 2.2 Testicular resident macrophages and local immune tolerance

In addition to the physical barrier, testicular immune privilege also relies on specialized local immune cell populations, among which testicular resident macrophages (tMφ) are the most representative. Unlike classical macrophages in peripheral inflamed tissues, tMφ under normal physiological conditions predominantly exhibit an immune–tolerant or M2–like phenotype, characterized by high expression of immunomodulatory molecules such as CD163 and CD206, constitutive secretion of anti–inflammatory cytokines including IL–10 and TGF–β, and relatively low expression of pro–inflammatory mediators such as TNF–α, IL–1β, and IL–6 (21, 22). This immunosuppressive microenvironment helps buffer cellular debris, leakage of autoantigens, and mild oxidative stress generated during normal spermatogenesis, thereby preventing persistent sterile inflammation in testicular tissue.

However, the immune–tolerant state of the testis is highly fragile. Under conditions of IR, toxicant exposure, or severe oxidative stress, large numbers of damaged cells release DAMPs, which can rapidly break the local immune balance and induce tMφ polarization toward a pro–inflammatory M1 phenotype (23). The large amounts of ROS, NO, and TNF–α released by M1 macrophages further weaken anti–ferroptosis defense systems such as GPX4/SLC7A11; on the other hand, lipid peroxidation products and DAMPs released from ferroptotic cells continuously activate local immune cells, amplifying the inflammatory effect. Therefore, in the context of testicular IR injury, immune imbalance, oxidative stress, and ferroptosis may not be independent of each other but rather interconnected and synergistic pathological networks that drive the collapse of the spermatogenic microenvironment (20, 24).

## 3 IR–induced ferroptosis in testicular cells

### 3.1 Lipid metabolism and iron metabolism: the metabolic basis of testicular ferroptosis

Testicular tissue, especially germ cells and mature spermatozoa, exhibits highly specialized lipid metabolic characteristics. Their cell membranes are rich in polyunsaturated fatty acids (PUFAs), particularly arachidonic acid (AA) and docosahexaenoic acid (DHA). These highly unsaturated lipids confer favorable membrane fluidity and membrane fusion capacity to spermatozoa, which are essential structural features for capacitation, the acrosome reaction, and fertilization (20). However, PUFAs are also the most vulnerable substrates for lipid peroxidation. Under the action of lipid remodeling enzymes such as ACSL4 and LPCAT3, PUFAs are continuously esterified and incorporated into membrane phospholipids, thereby significantly increasing the sensitivity of testicular tissue to oxidative stress and ferroptosis. When ROS accumulate extensively, these PUFA–rich membrane structures are highly prone to lipid peroxidation chain reactions, ultimately inducing cell membrane disruption and ferroptosis.

At the same time, testicular spermatogenesis is highly dependent on iron metabolism. Key processes such as DNA replication, mitochondrial respiratory chain function, and ribonucleotide reductase activity all require iron as a catalyst. Sertoli cells establish a locally refined iron homeostasis regulatory system through transferrin, ferritin, and related iron transporters, ensuring the iron supply needed for the continuous proliferation and differentiation of germ cells (18, 25, 26). However, this metabolic combination of “high iron demand – high PUFA enrichment” also renders the testis a tissue highly susceptible to ferroptosis. If radiation perturbs iron storage and transport in Sertoli cells, expansion of the labile iron pool could enhance Fenton chemistry and lipid peroxidation, thereby increasing ferroptotic susceptibility within the seminiferous epithelium. Thus, the testis-specific lipid profile and iron metabolic features are not only the physiological basis for normal reproductive function but also key factors underlying oxidative damage and ferroptosis upon radiation exposure.

### 3.2 Candidate molecular mechanisms linking IR to testicular ferroptosis

Precisely because the testicular microenvironment possesses the unique characteristics of high PUFA accumulation and high iron dependency, the damage caused by IR exhibits distinct pathological features. These mechanisms can be summarized into four interconnected dimensions: iron homeostasis disruption, lipid peroxidation amplification, antioxidant defense failure, and mitochondrial/FSP1 pathway dysfunction.

#### 3.2.1 IR and ROS-driven lipid peroxidation chain reaction

The plasma membranes of testicular germ cells are rich in PUFAs, rendering them naturally susceptible to oxidative damage. When IR penetrates testicular tissue, it not only directly contributes to macromolecular breakage such as DNA damage, but also instantaneously generates large amounts of highly toxic reactive oxygen species (ROS), such as hydroxyl radicals (·OH), through water radiolysis (11). This exogenous and severe oxidative stress directly overwhelms the cellular basal antioxidant defenses and, using free iron as a catalyst, attacks PUFAs on the cell membrane, triggering an uncontrolled lipid peroxidation chain reaction (27, 28). This process may provide an early physicochemical route capable of initiating lipid peroxidation and increasing ferroptotic susceptibility within the seminiferous epithelium.

#### 3.2.2 Dysregulation of iron metabolism networks and autophagy-dependent iron overload

Iron, as a key trace element, participates in the regulation of male reproductive function and exerts dual effects on testicular tissue. Appropriate iron content is necessary for maintaining testosterone synthesis and spermatogenesis. However, iron overload can lead to male reproductive dysfunction by triggering testicular oxidative stress, lipid peroxidation, and even testicular ferroptosis (29). Beyond direct ROS generation, perturbation of iron homeostasis represents a plausible additional route through which IR may increase ferroptotic susceptibility. NCOA4–mediated ferritinophagy provides a mechanistic basis for increasing the labile iron pool by promoting ferritin degradation and Fe²^+^ release. If excessively activated following irradiation, this process could enhance Fenton chemistry and lipid peroxidation in testicular cells (14). On the other hand, radiation–induced inflammatory signaling may potentially influence iron regulatory networks, including the balance between iron uptake and export pathways involving transferrin receptor 1 (TfR1) and ferroportin (FPN). However, whether such alterations occur in irradiated testicular tissue remains to be directly determined.

#### 3.2.3 p53-mediated repression of the SLC7A11/GPX4 antioxidant axis

IR-induced DNA double-strand breaks (DSBs) rapidly activate the intracellular DNA damage response (DDR) network, in which the tumor suppressor p53 serves as a key connector linking radiation injury to metabolic remodeling. Studies have shown that upon intense radiation stress, highly activated nuclear p53 induces ferroptosis through two pathways (30). First, it directly suppresses SLC7A11 gene expression, blocking cystine uptake mediated by the Xc^-^ system. Downregulation of System Xc^-^ pathway leads to a forced interruption of cystine uptake in testicular cells (especially spermatogenic cells), which in turn causes rapid glutathione (GSH) depletion and inactivation of GPX4 activity (13). Second, p53 activates SAT1 to promote polyamine metabolism, accelerating lipoxygenase-dependent lipid peroxidation (31). Deprived of the reductive protection of GPX4, the large amounts of lipid peroxides induced by radiation cannot be cleared and directly execute cell membrane perforation and collapse.

#### 3.2.4 Mitochondrial dysfunction and compensatory failure of the FSP1 pathway

As an important regulator of ferroptosis, mitochondrial dynamics and dysfunction are involved throughout the progression of ferroptotic cell death (32). Following these physical changes in mitochondrial membrane architecture, sustained dissipation of the transmembrane potential (ΔΨm) may impair electron transport chain (ETC) function, contributing to electron leakage and excessive ROS accumulation (33).

In this process, mitochondria serve not only as a major source of ROS but also as an important site for lipid peroxidation amplification. Ferroptosis suppressor protein 1 (FSP1) provides a GPX4–independent defense pathway by regenerating reduced CoQ10 (ubiquinol) from ubiquinone, thereby suppressing lipid peroxidation and limiting lipid radical propagation on cellular membranes (34, 35). Mitochondrial dysfunction may amplify ROS production and lipid peroxidation, whereas the FSP1–CoQ10 axis may counteract this process by limiting lipid radical propagation (36) (**Figure 3**).

**Figure 3:**
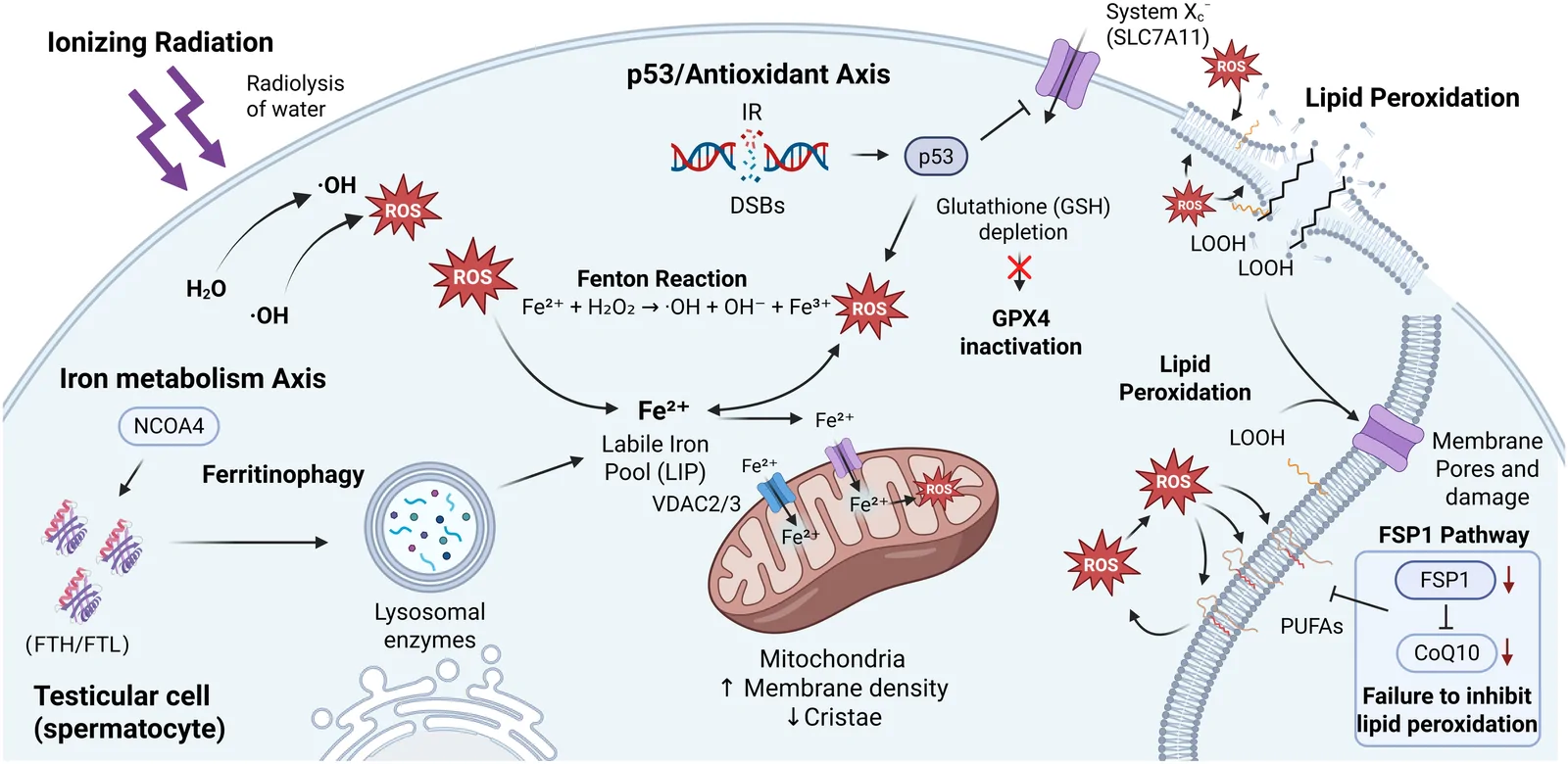
Proposed molecular mechanisms linking ionizing radiation (IR) to ferroptosis in testicular cells. IR exposure triggers water radiolysis to generate hydroxyl radicals (·OH) and induces DNA double-strand breaks (DSBs) that activate the p53 pathway. In the iron metabolism axis, NCOA4-mediated ferritinophagy promotes the degradation of ferritin (FTH/FTL) by lysosomal enzymes, releasing abundant Fe²^+^ into the labile iron pool (LIP). This free iron is transported into mitochondria via VDAC2/3, driving the Fenton reaction (Fe²^+^ + H_2_O_2_ → ·OH + OH^-^ + Fe³^+^), which exacerbates ROS production, and induces characteristic mitochondrial structural changes (increased membrane density and reduced cristae). Meanwhile, in the p53/antioxidant axis, activated p53 transcriptionally inhibits the System Xc^-^ core subunit SLC7A11, leading to glutathione (GSH) depletion and subsequent inactivation of GPX4. Furthermore, downregulation of the FSP1 pathway (with decreased CoQ10 levels) results in a failure to inhibit lipid peroxidation. Collectively, the convergence of iron overload, ROS accumulation, and impaired GPX4- and FSP1-dependent antioxidant defenses could promote severe lipid peroxidation of membrane PUFAs and thereby increase susceptibility to ferroptotic membrane damage. Some graphical elements were created with BioRender.com.

## 4 Crosstalk between ferroptosis and immune response in the testis

IR-induced injury is not merely manifested as the loss of a single germ cell type, but rather as a systemic functional decline driven by cellular heterogeneity. Here, ferroptosis–immune crosstalk refers to the bidirectional regulation between ferroptotic stress and immune remodeling, including DAMP–mediated macrophage activation, inflammatory cytokine signaling, and immune–mediated suppression of antioxidant defenses such as the SLC7A11–GSH–GPX4 axis. To clarify how these cell–specific events may interact, **Figure 1** integrates these relationships into a testable conceptual framework linking ferroptotic injury, BTB dysfunction, innate and adaptive immune activation, and secondary tissue damage. Available evidence suggests that Sertoli cells, Leydig cells, and immune cells may interact within a proposed “ferroptosis–immune response” crosstalk network that could amplify radiation–induced testicular injury.

### 4.1 Proposed links between Sertoli cell ferroptosis, BTB dysfunction, and autoimmune inflammation

Under radiation stress, Sertoli cells may represent important targets of ferroptotic injury, and impairment of their metabolic and barrier–supporting functions could contribute to BTB dysfunction (19, 25).

At the molecular and metabolic levels, evidence from testicular injury and ferroptosis models indicates that alterations in GPX4, ACSL4/LPCAT3, and NCOA4–mediated ferritinophagy are associated with increased ferroptotic susceptibility. These pathways provide plausible mechanisms through which radiation–induced oxidative stress could promote iron overload, lipid peroxidation, and ferroptotic injury in Sertoli cells (37). The depletion of GSH and inactivation of GPX4 lead to excessive release of lipid ROS from mitochondria, triggering severe ferroptosis. This primarily manifests as a reduction in Sertoli cell number and a systemic collapse of their nurturing function: mitochondrial dysfunction leads to a sharp drop in ATP synthesis, which in turn impairs the ability of Sertoli cells to take up glucose and synthesize lactate, disrupting the metabolic coupling and physical support for spermatogonial stem cells (SSCs) and thereby causing loss of their capacity to maintain SSC self-renewal and differentiation (25).

Ferroptosis–associated lipid peroxidation and oxidative stress provide several plausible mechanisms through which Sertoli–cell junctional integrity could be compromised (37, 38). On the one hand, lipid peroxidation products such as 4-HNE interfere with the synthesis and secretion of glial cell line-derived neurotrophic factor (GDNF) and stem cell factor (SCF), disrupting the SSC microenvironment (39); meanwhile, oxidative damage to the cytoskeletal proteins of Sertoli cells causes retraction of their cytoplasmic processes, directly weakening the stability of cell-cell junctions (37). On the other hand, reactive lipid products such as 4-HNE directly oxidatively modify and degrade tight junction proteins including Occludin, Claudin-11, and ZO-1; moreover, the excessive accumulation of ROS further suppresses the expression of these tight junction proteins at the transcriptional level by activating signaling pathways such as p38 MAPK and NF-κB, potentially contributing to progressive loss of BTB structural integrity (40).

These observations support the possibility that BTB dysfunction and local inflammatory responses may become mutually reinforcing. DAMPs released from injured or ferroptotic cells may favor pro–inflammatory macrophage activation, resulting in increased production of cytokines such as TNF–α, IL–1β, and IL–6 (41). These inflammatory mediators not only enhance vascular permeability but also may act directly on Sertoli cells via paracrine signaling, inducing cytoskeletal rearrangement and junctional disassembly (42); among them, TNF-α activates myosin light chain kinase (MLCK) in Sertoli cells, leading to cell contraction and junctional relaxation, thereby further widening BTB gaps and significantly accelerating barrier function loss (43).

Crucially, BTB rupture breaks the original “immune privilege” status of the testis and shifts it toward a pathological state of “adaptive immune activation” (44). Autoantigens that were originally physically isolated become exposed in the testicular interstitium, where they are taken up by dendritic cells and macrophages and presented, subsequently activating peripheral T lymphocytes and inducing infiltration of autoreactive CD4^+^ and CD8^+^ T cells into testicular tissue (45). Activated T cells further secrete cytokines such as IFN-γ, exacerbating local inflammation and directly attacking surviving spermatogenic cells through cytotoxic effects (46).

Taken together, these observations support a proposed positive–feedback model in which Sertoli cell ferroptotic injury may compromise BTB integrity, facilitate the exposure of germ–cell antigens, and promote inflammatory and adaptive immune responses that could further lower the ferroptosis threshold in neighboring cells (15). If sustained *in vivo*, such a loop could contribute to persistent seminiferous epithelial injury and delayed recovery after irradiation. However, direct longitudinal evidence demonstrating the complete sequence from Sertoli cell ferroptosis to BTB disruption, adaptive immune activation, and secondary ferroptosis in irradiated testes remains limited. We therefore regard the “Sertoli cell–BTB–immune” loop as a testable mechanistic framework rather than an established causal pathway. From this perspective, preservation of Sertoli cell homeostasis and BTB integrity represents a candidate strategy for interrupting the proposed amplification process.

### 4.2 Functional “delayed injury” of Leydig cells

In parallel with the proposed Sertoli cell–BTB axis, delayed Leydig cell dysfunction may represent another component of the long–term testicular response to radiation. Although they have a strong tolerance to acute apoptosis, their active mitochondrial metabolism and high lipid content render them highly sensitive to chronic ferroptosis (47). Radiation-induced ferroptosis and lipid peroxidation disrupt the mitochondrial membrane potential (ΔΨm) and ATP production capacity of Leydig cells, thereby interfering with cholesterol transport into the inner mitochondrial membrane, inhibiting the function of steroidogenic acute regulatory protein (StAR), and attenuating the catalytic activity of 3β-hydroxysteroid dehydrogenase (3β-HSD) (48, 49).

This iron-overload- and lipid-peroxidation-driven impairment of androgen synthesis leads to a long-term decline in serum testosterone levels, disrupts the negative feedback regulation of the hypothalamic-pituitary-testicular (HPT) axis, and results in elevated luteinizing hormone (LH) and follicle-stimulating hormone (FSH) that nevertheless fail to restore peripheral androgenic function (47, 50). Consequently, the functional delayed injury of Leydig cells not only delays long-term repair of the germinal epithelium but also persistently impedes the regeneration of spermatogonial stem cells (SSCs), thereby exacerbating the overall functional decline of the testicular microenvironment (51, 52). Moreover, although Leydig cell ferroptosis primarily manifests as metabolic and endocrine dysfunction, their lipid peroxidation and membrane damage may also promote the release of DAMPs, thereby mildly activating resident macrophages in the local microenvironment, increasing pro-inflammatory cytokine levels, and amplifying the vicious cycle of Sertoli cell ferroptosis and immune responses (47). Thus, ferroptosis–associated metabolic and functional impairment of Leydig cells may represent a potential contributor to long–term dysregulation of the testicular microenvironment after radiation.

### 4.3 DAMP release mediated by ferroptosis and immune remodeling of macrophages

IR-induced ferroptosis has significant pro-inflammatory characteristics. The lipid peroxidation and structural disruption of the cell membrane caused by ferroptosis promote the release substantial amounts of DAMPs, thereby remodeling the local testicular immune microenvironment.

#### 4.3.1 Cascade release of DAMPs

Unlike apoptosis, in which cell membrane integrity is relatively preserved, the characteristic membrane lipid peroxidation of ferroptosis leads to progressive loss of membrane integrity and eventual plasma membrane rupture. This process triggers the massive release of endogenous signaling molecules such as high-mobility group box 1 (HMGB1), mitochondrial DNA (mtDNA), and heat shock proteins (HSPs) (17). Among them, HMGB1 can translocate from the nucleus to the cytoplasm under cellular stress conditions and is subsequently released into the extracellular space upon loss of plasma membrane integrity. Studies by Miyake et al. have shown that lipid peroxidation-induced nuclear membrane damage during ferroptosis can occur prior to plasma membrane rupture, thereby promoting passive release of HMGB1 from the nucleus to the cytoplasm (53). This intracellular translocation is regulated by ROS and the RAS-JNK/p38 MAPK signaling cascade. This ROS-dependent translocation promotes autophagic flux by dissociating the BECN1/Bcl-2 complex, thereby facilitating autophagy, ferritinophagy, and ferroptosis (54). HMGB1, as a classic DAMP, activates immune responses through interactions with the receptor for advanced glycation end products (RAGE, also known as AGER) and Toll-like receptors (TLRs) 2 and 4. Therefore, inhibiting HMGB1 release or RAGE deficiency attenuates the ferroptosis-induced inflammatory response of macrophages (17, 55).

#### 4.3.2 DAMP-driven M1 polarization of macrophages and ferroptosis positive feedback

Extracellular DAMPs activate Toll-like receptor 4 (TLR4) and RAGE on the macrophage surface, triggering robust activation of downstream NF-κB and MAPK signaling pathways. This signaling cascade induces macrophage phenotypic remodeling toward a pro-inflammatory M1 type, leading to massive secretion of tumor necrosis factor-α (TNF-α) and nitric oxide (NO) (56). This local inflammatory microenvironment results in immune cell-derived strong oxidants (including NO and its reactive nitrogen intermediates) as well as pro-inflammatory factors such as TNF-α not only targeting the clearance of pathological cells but also non-specifically attacking adjacent healthy germ cell membranes, inducing lipid peroxidation and membrane structural damage. TNF–α, NO, and other macrophage–derived inflammatory mediators have been reported to impair cellular antioxidant defenses in several experimental contexts. Whether similar mechanisms operate in irradiated Sertoli cells requires direct validation. We therefore propose that DAMP–driven macrophage activation could lower the ferroptosis threshold in neighboring Sertoli cells, which may in turn release additional inflammatory signals and thereby form a candidate ferroptosis–immune amplification loop (57) (**Figure 1**).

## 5 Interplay network between ferroptosis and other programmed cell death modalities

In IR–induced testicular injury, ferroptosis may not occur in isolation but could intersect with apoptosis, autophagy, pyroptosis, and necroptosis through shared mediators such as oxidative stress, mitochondrial dysfunction, DAMP release, and inflammatory signaling (58).

### 5.1 Synergistic interaction between ferroptosis and apoptosis

Apoptosis has long been recognized as the classical cell death mode underlying IR-induced testicular germ cell injury, primarily functioning to eliminate cells with irreparable DNA damage to maintain spermatogenic homeostasis (59). However, accumulating evidence indicates significant cross-regulation between ferroptosis and apoptosis (60). p53 is considered an important molecular hub connecting these two processes. On the one hand, IR-induced DNA double-strand breaks activate p53-dependent apoptotic pathways, triggering mitochondrial apoptosis by upregulating pro-apoptotic factors such as Bax and Puma; on the other hand, activated p53 also inhibits SLC7A11 expression, impairing cystine uptake mediated by System Xc^-^ pathway, leading to GSH depletion and decreased GPX4 activity, thereby promoting lipid peroxidation and ferroptosis (61, 62).

Furthermore, lipid peroxidation end products (e.g., 4-HNE) can not only directly damage cell membrane structures but also amplify mitochondrial dysfunction and promote Caspase activation, forming an apoptosis-ferroptosis amplification loop (62). Radiation injury may involve dynamic interactions between apoptosis and ferroptosis.

### 5.2 Synergistic effect of autophagy-dependent ferroptosis

Autophagy plays a dual role in testicular homeostasis. Basal autophagy helps clear damaged organelles and maintain metabolic balance, whereas under persistent IR stimulation, excessive autophagy may promote ferroptosis. Among the underlying mechanisms, NCOA4-mediated ferritinophagy is considered a key bridge connecting autophagy and ferroptosis (63). NCOA4 promotes ferritin degradation and releases large amounts of Fe²^+^, leading to expansion of the labile iron pool (LIP), sustained enhancement of the Fenton reaction, and accumulation of lipid ROS, thereby inducing ferroptosis. Because Sertoli cells are responsible for iron transport and metabolic buffering within the testis, they are particularly sensitive to ferritinophagy-induced ferroptosis (14, 64).

Evidence from autophagy, ferroptosis, and immune studies suggests several potential routes through which dysregulated autophagy could connect intracellular ferroptotic stress with testicular immune remodeling. Secretory autophagy may facilitate extracellular release of DAMPs such as HMGB1 (55, 65), whereas defective mitochondrial quality control could favor mtDNA accumulation and activation of inflammatory signaling pathways such as cGAS–STING (66, 67). In parallel, iron handling and ferritinophagy in macrophages may influence inflammasome activation and inflammatory polarization. These observations provide mechanistic plausibility for an autophagy–ferroptosis–immune axis; however, the complete sequence has not been directly demonstrated in irradiated testicular tissue and should therefore be considered a proposed component of the present framework.

### 5.3 Potential inflammatory crosstalk between ferroptosis and pyroptosis/necroptosis

Under IR conditions, cell membrane rupture caused by ferroptosis releases large amounts of DAMPs and lipid metabolites, which not only activate tMφ but also induce NLRP3 inflammasome activation in Sertoli cells (68, 69). Meanwhile, TNF-α, NO, and ROS released by M1 macrophages can further suppress GPX4-dependent antioxidant defenses, enhancing the sensitivity of surrounding cells to ferroptosis. Together, these interactions could establish a proposed feedback loop of “ferroptosis → inflammatory activation → secondary ferroptosis,” potentially exacerbating BTB dysfunction and local immune imbalance (15, 58).

### 5.4 Potential immunomodulatory implications of cell–death mode switching

In IR-induced testicular injury, different programmed cell death (PCD) modalities do not simply occur in parallel; instead, they are dynamically regulated by the local immune microenvironment. In the early stage of injury, lower levels of DNA damage typically activate p53-dependent apoptosis, clearing damaged cells in a relatively “immunologically silent” manner. As ROS accumulation, lipid peroxidation, and mitochondrial dysfunction progressively worsen, the SLC7A11/GPX4 axis becomes increasingly depleted, and the cell death mode shifts toward a ferroptotic phenotype (70).

At the same time, DAMPs released from ferroptotic cells activate TLR4/NF-κB and NLRP3 inflammasome signaling, driving the local immune status from immune tolerance toward pro-inflammatory activation (17). M1 macrophages further release TNF-α, IL-1β, NO, and ROS, continuously lowering the ferroptosis threshold in neighboring cells and inducing pyroptosis and necroptosis.

On the basis of these overlapping signaling features, we propose that severe radiation stress could generate a PANoptosis–like inflammatory death network involving ferroptosis, pyroptosis, and necroptosis (71). Within this conceptual model, Sertoli cells and the BTB may function as important spatial hubs linking cellular injury to immune activation. However, direct evidence demonstrating coordinated PANoptosis–like signaling in irradiated testicular tissue is currently lacking (72). Thus, this model should be considered a hypothesis for future experimental testing rather than a defined mode of testicular cell death after IR.

## 6 Potential therapeutic strategies targeting ferroptosis and immunomodulation

Given the potential contribution of ferroptosis to ionizing radiation–induced injury of the seminiferous epithelium and Sertoli cells, targeting ferroptosis–related pathways has emerged as a candidate strategy for reproductive radioprotection. However, the therapeutic relevance of these strategies should be interpreted cautiously. Most ferroptosis–targeting interventions in the context of testicular injury remain supported primarily by experimental models, and their efficacy, optimal timing, and long–term reproductive safety have not been fully established. In particular, differences in radiation dose, exposure patterns, disease stage, and species–specific reproductive physiology may substantially influence treatment responses. Moreover, ferroptosis is closely interconnected with immune regulation and tissue repair; therefore, isolated inhibition of ferroptosis–related pathways may not be sufficient once inflammatory amplification or blood–testis barrier disruption has occurred. Future studies should clarify the therapeutic window, cell–type specificity, and potential risks associated with long–term modulation of ferroptosis–related pathways. Moreover, whether these interventions can achieve sufficient exposure in specific testicular compartments, particularly Sertoli cells protected by the blood–testis barrier, remains an important translational challenge. As summarized in **Table 1**, the relevant intervention strategies can be divided into two categories: clinically accessible drugs/antioxidant combinations and candidate strategies still at the experimental research stage. The former include melatonin, edaravone, and clinically used antioxidant combinations such as NAC plus selenium, CoQ10 plus L-carnitine. Studies have shown that melatonin can reduce testicular oxidative stress and ferroptosis-related damage by activating the NRF2/GPX4 axis, inhibiting mitochondrial ROS generation, lowering lipid peroxidation, and modulating inflammatory responses (85). Edaravone, a free radical scavenger, exerts anti-ferroptotic effects by inhibiting lipid ROS accumulation and regulating the NRF2/GPX4 signaling pathway, showing potential protective effects in testicular ischemia-reperfusion and toxin-induced injury models (99). In addition, clinically applicable ferroptosis-related antioxidant combinations such as NAC plus selenium, and CoQ10 plus L-carnitine can improve semen quality and protect germ cells by supplementing the GSH-GPX4 antioxidant system, enhancing the mitochondrial antioxidant network, and alleviating oxidative-inflammatory damage (95–98).

**Table 1:** Therapeutic agents targeting ferroptosis and inflammation.

Therapeutic strategy	Representative agents	Primary target/pathway	Mechanism	Protective effects on testis	Evidence status/translational stage	References
Iron chelators	Deferoxamine (DFO), Deferiprone (DFP)	Labile iron pool/Fenton reaction	Chelate Fe²^+^, inhibit ROS amplification and lipid peroxidation	Reduce Sertoli cell ferroptosis, protect spermatogenesis and blood-testis barrier integrity	Clinically approved for iron overload disorders; application in radiation-induced testicular injury remains preclinical	(73)
Lipophilic radical scavengers	Ferrostatin-1 (Fer-1), Liproxstatin-1 (Lip-1)	Lipid ROS/membrane phospholipids	Scavenge lipid radicals, terminate lipid peroxidation chain reaction	Reduce germ cell loss, mitochondrial damage, and membrane oxidative injury	Experimental ferroptosis inhibitors; no clinical application currently	(74, 75)
GPX4 restoration	Selenium, L-selenomethionine	GPX4-dependent antioxidant defense	Enhance GPX4 synthesis and detoxification of phospholipid hydroperoxides	Improve sperm quality and antioxidant capacity	Nutritional compounds with clinical availability; ferroptosis-targeting application requires further validation	(76, 77)
System Xc^-^ activation	N-acetylcysteine (NAC), Sulforaphane	SLC7A11/GSH biosynthesis	Restore cysteine uptake and intracellular GSH pool, support GPX4 activity	Enhance endogenous antioxidant capacity, inhibit ferroptosis	NAC clinically available; protective effects against radiation-induced testicular injury remain insufficiently validated	(78)
NRF2 activators	Sulforaphane, Bardoxolone methyl	NRF2/Keap1 signaling pathway	Upregulate antioxidant and iron-related genes (SLC7A11, FTH1, HO-1, GCLC)	Protect redox homeostasis in Leydig and Sertoli cells	Mostly preclinical; clinical translation for reproductive protection remains unclear	(79, 80)
Ferritinophagy inhibition	3-Methyladenine (3-MA), autophagy inhibitors	NCOA4-mediated ferritinophagy	Prevent ferritin degradation and excessive free iron release	Reduce iron overload and secondary ferroptosis	Experimental strategy; clinical applicability remains limited	(81)
Mitochondrial protection	Coenzyme Q10 (CoQ10), MitoQ	Mitochondrial ROS/membrane potential	Stabilize mitochondrial membrane integrity, inhibit mitochondrial lipid peroxidation	Improve ATP production, sperm motility, and Leydig cell steroidogenesis	CoQ10 is clinically available as a supplement; ferroptosis-related reproductive protection remains investigational	(82–84)
Melatonin-mediated ferroptosis inhibition	Melatonin	NRF2/GPX4 axis, mitochondrial ROS, inflammatory signals	Enhance antioxidant defense, inhibit lipid peroxidation and ferroptosis, modulate immune responses	Protect spermatogenic cells, alleviate testicular injury induced by heat stress, chemotherapy, and ischemia	Clinically available compound; radioprotective effects in testicular injury remain preclinical	(85)
Anti-inflammatory intervention	NSAIDs, TNF-α inhibitors, Pentoxifylline	TNF-α/NF-κB signaling pathway	Inhibit inflammatory amplification triggered by ferroptosis-associated DAMPs	Reduce blood-testis barrier disruption and inflammatory damage	Clinically available drugs; application in ferroptosis-associated testicular injury remains preclinical	(86, 87)
HMGB1 blockade	Anti-HMGB1 antibody, RAGE/TLR4 antagonists	HMGB1-TLR4/RAGE axis	Inhibit DAMP-mediated macrophage activation and cytokine release	Prevent progression of chronic sterile inflammation and autoimmune orchitis	Experimental strategy; clinical application for testicular injury remains unavailable	(88)
Macrophage reprogramming	IL-10, MSC-derived exosomes	M1/M2 polarization	Promote anti-inflammatory M2 phenotype, suppress cytokine storm	Restore immune privilege, stabilize testicular microenvironment	Emerging immunomodulatory strategy; currently limited to preclinical studies	(89)
Natural antioxidants	Curcumin, Quercetin, Lycopene, Vitamins C/E	ROS and lipid peroxidation	Neutralize ROS, improve endogenous antioxidant enzyme activities	Protect sperm DNA integrity, improve semen parameters	Widely available compounds; protective effects against ferroptosis-related injury remain mainly preclinical	(90–94)
Clinically available ferroptosis-related antioxidant combinations	NAC + selenium, CoQ10 + L-carnitine	GSH-GPX4 system, mitochondrial antioxidant network	Synergistically inhibit lipid peroxidation and oxidative-inflammatory damage	Improve semen quality, protect germ cells from oxidative injury	Clinically accessible combinations; application in radiation-induced ferroptosis remains investigational	(95–98)
Drug repurposing (ferroptosis inhibitor)	Edaravone	Lipid ROS, NRF2/GPX4 signaling pathway	Potent free radical scavenger, inhibits lipid peroxidation and ferroptosis	Potential protective effect against testicular ischemia-reperfusion and toxin-induced injury	Repurposed compounds with preclinical evidence; translational potential requires further validation	(99)
Stem cell/exosome therapy	MSC-derived exosomes	Tissue repair and immunomodulation	Deliver anti-inflammatory miRNAs and antioxidant proteins	Promote blood-testis barrier repair and regeneration of spermatogenic microenvironment	Promising regenerative approach; currently restricted to preclinical studies	(100)
Nanomedicine delivery	Lipid nanoparticles, ROS-responsive nanocarriers	Targeted ferroptosis modulation	Improve bioavailability and testicular targeting of ferroptosis inhibitors	Enhance therapeutic precision, reduce systemic toxicity	Early translational strategy; mainly supported by preclinical evidence	(101, 102)
Combination therapy	Fer-1 + anti-inflammatory drugs; NAC + selenium; CoQ10 + antioxidants	Ferroptosis-immune amplification loop	Simultaneously block lipid peroxidation and inflammatory signals	More effectively restore blood-testis barrier integrity and fertility potential	Emerging strategy; efficacy and clinical translation remain to be established	(103, 104)

In contrast, specific ferroptosis inhibitors, ferritinophagy inhibitors, HMGB1/RAGE/TLR4 blockers, stem cell/exosome therapies, and nanodelivery systems are mostly still at the basic research or animal experiment stage and have not yet become standard clinical medications for testicular radioprotection. The following sections describe these non-clinical or translational-stage strategies targeting ferroptosis and immunomodulation.

To directly connect these therapeutic strategies with the proposed pathogenic framework, **Figure 2** maps candidate interventions to specific nodes within the ferroptosis–immune network. These nodes include labile iron/Fenton chemistry, lipid peroxidation, the SLC7A11–GSH–GPX4 and FSP1–CoQ10 antioxidant systems, NCOA4–mediated ferritinophagy, DAMP–associated HMGB1–RAGE/TLR4 signaling, pro–inflammatory macrophage activation, and downstream BTB repair. Importantly, these intervention points differ substantially in the maturity and directness of supporting evidence; therefore, **Figure 2** should be interpreted as a map of candidate therapeutic targets rather than as a clinically validated treatment algorithm for radiation–induced testicular injury.

### 6.1 Ferroptosis–immune–testicular damage axis: a proposed integrative framework

Although ferroptosis has traditionally been interpreted as an intracellular metabolic disorder characterized by iron accumulation, lipid peroxidation, and antioxidant system failure, emerging evidence suggests that its pathological consequences may extend beyond individual cell death. In the irradiated testis, ferroptotic injury may serve as an initiating or amplifying event that connects cellular metabolic stress with immune remodeling and tissue dysfunction. Specifically, radiation–induced oxidative and iron–dependent stress may increase ferroptotic susceptibility in Sertoli cells and germ cells, leading to lipid peroxidation, DAMP release, and disruption of the BTB. These events may subsequently activate innate and adaptive immune responses, including macrophage polarization and T–cell–mediated inflammation, which could further compromise antioxidant defenses and increase ferroptotic vulnerability in neighboring cells. Therefore, we propose a ferroptosis–immune–testicular damage axis as a hypothesis–generating framework to integrate currently fragmented observations and provide a conceptual basis for future mechanistic validation and therapeutic intervention.

### 6.2 Ferroptosis–targeting strategies under investigation

#### 6.2.1 Iron chelators

Iron chelators have already been clinically applied for the management of iron overload disorders, particularly transfusion–dependent anemias and other conditions associated with secondary iron accumulation (105, 106). However, their established clinical indications mainly focus on systemic iron burden reduction, whereas their potential application in radiation–induced testicular injury remains investigational. Therefore, their reproductive safety, optimal dosing regimen, and ability to achieve effective concentrations within the testicular microenvironment require further evaluation.

Targeting and reducing free iron in the labile iron pool is a key driver for catalyzing radiation-induced ROS burst and lipid peroxidation. Iron chelators (e.g., deferoxamine, DFO, and deferiprone, DFP) can directly bind free ferrous ions in the cytoplasm of testicular cells, thereby blocking the Fenton reaction at its source (12). Studies have shown that administration of iron chelators before or early after radiation exposure has been reported to reduce the excess iron released from radiation-induced ferritinophagy in Sertoli cells, reduce lipid peroxidation levels, and significantly improve post-irradiation germ cell apoptosis and spermatogenesis (107).

#### 6.2.2 Lipophilic radical scavengers block the lipid peroxidation chain

Targeting the physicochemical process by which radiation triggers large-scale oxidation of polyunsaturated fatty acids (PUFAs) in testicular cell membranes, lipophilic antioxidants show radioprotective potential. The specific ferroptosis inhibitors Ferrostatin–1 (Fer–1) and Liproxstatin–1 (Lip–1) specifically reside in the lipid bilayers of cell and mitochondrial membranes. When ionizing radiation initiates primary lipid radicals, these small–molecule inhibitors act as radical-trapping antioxidants (RTAs), directly neutralizing lipid peroxides (PLOOH) by donating hydrogen atoms, thereby forcibly terminating the chain amplification of lipid peroxidation and protecting the integrity of germ cell membranes (108, 109).

#### 6.2.3 Activation and restoration of the endogenous SLC7A11/GPX4 antioxidant axis

Besides directly scavenging free radicals, pharmacologically reshaping the local endogenous antioxidant defense system in the testis is a more durable protection strategy. To counteract radiation-induced p53 activation leading to repression of the SLC7A11/GPX4 axis, current research focuses on NRF2 agonists or positive regulators of System Xc^-^ (110, 111). These agents can competitively relieve transcriptional repression of the SLC7A11 gene, restore cysteine uptake capacity, promote GSH biosynthesis, and thereby reactivate GPX4 enzymatic activity, fundamentally reinstating the resilience of the spermatogenic microenvironment against radiation-induced oxidative stress (112).

Nevertheless, the majority of ferroptosis–specific inhibitors remain restricted to preclinical studies. Their potential clinical application requires further evaluation regarding pharmacokinetic properties, reproductive toxicity, ability to reach testicular compartments, and the optimal timing of intervention after radiation exposure.

### 6.3 Natural products and trace elements targeting ferroptosis

In addition to specific ferroptosis inhibitors, antioxidants can eliminate ROS and activate other pathways to prevent ferroptosis. The glutathione precursor N-acetylcysteine is a widely used antioxidant, and studies have shown that the NRF2 inducer sulforaphane improves sperm concentration and quality in busulfan-treated mice (107). Various natural compounds such as quercetin, GSH, vitamin C, vitamin B9, carotenoids, and curcumin have also been reported to protect the testis from injury and improve semen quality (113–115).

The trace element selenium (Se) is deeply linked to resistance to ferroptosis. GPX4 is a typical selenoprotein; its catalytic activity for detoxifying lipid hydroperoxides strictly depends on the selenocysteine (Sec) residue at its active site (116). After ionizing radiation, the testis faces extreme oxidative stress, leading to rapid depletion of endogenous GSH and overload of GPX4. Studies indicate that early dietary intervention or systemic supplementation with organic selenium (e.g., L-selenomethionine) may enhance GPX4–related antioxidant capacity; however, whether this approach provides sufficient protection against radiation–induced ferroptotic injury in the testis remains to be determined. (117).

### 6.4 Blockade of immune inflammatory amplification and combination intervention strategies

Given the proposed positive–feedback interaction between ferroptosis and pro–inflammatory macrophage activation, targeting ferroptosis alone may be insufficient once broader immune dysregulation has developed. Accordingly, combined ferroptosis– and inflammation–directed interventions may warrant further investigation. For example, one study demonstrated that targeting the core DAMPs released during ferroptosis with anti-HMGB1 neutralizing antibodies or RAGE/TLR4 antagonists effectively cuts off the secretion of pro-inflammatory cytokines, prevents their transmission to the immune system, and alleviates inflammation in a rat model of autoimmune orchitis (88). Moreover, combining classical anti-inflammatory drugs (such as NSAIDs or specific cytokine inhibitors) with ferroptosis inhibitors may rescue damaged Sertoli cells, accelerate structural repair of the blood-testis barrier, and re-establish testicular immune privilege homeostasis (**Table 1**). However, immune–targeted interventions require careful consideration because excessive suppression of inflammatory responses may interfere with physiological immune surveillance and tissue repair processes within the testicular microenvironment.

## 7 Discussion

In recent years, ferroptosis has garnered extensive academic attention as a key pathophysiological hub linking oxidative stress, metabolic disorders, and immune responses (118). In the context of ionizing radiation (IR)–induced testicular injury, the ferroptosis hypothesis has provided a potential theoretical dimension for elucidating the mechanisms of radiation damage to the male reproductive system (119). However, current studies are mostly limited to descriptive associations; systematic and causal evidence is still lacking regarding the precise spatiotemporal localization, kinetic characteristics, and interplay with the local immune microenvironment of ferroptosis in testicular radiation pathology. In this review, the term “ferroptosis–immune crosstalk framework” is used to describe a hypothesis–generating model rather than an established causal pathway. The framework integrates evidence from radiation biology, testicular ferroptosis, BTB and immune–privilege studies, and ferroptosis–immune interactions reported in other tissues. Accordingly, mechanistic links supported only indirectly are interpreted as proposed relationships, whereas direct evidence from irradiated testicular models is distinguished where available. This distinction is particularly important because several key steps in the proposed network have not yet been demonstrated within a single spatiotemporally resolved experimental system. Based on existing evidence, this discussion focuses on several unresolved controversies and frontier issues in the field.

Existing models of ferroptosis–associated tissue injury have primarily focused on intracellular metabolic disturbances, including iron accumulation, lipid peroxidation, mitochondrial dysfunction, and antioxidant system failure (7, 12). In parallel, ferroptosis–immune interaction models developed mainly in cancer and inflammatory diseases have emphasized the role of ferroptotic cells as sources of immunogenic signals, such as DAMPs, that reshape immune responses (120). However, these models do not fully consider the unique characteristics of the testicular microenvironment, particularly the immune–privileged status, the structural role of Sertoli cells in maintaining the BTB, and the functional coordination between somatic cells and germ cells. In this review, we extend previous ferroptosis–centered models by proposing a testis–specific ferroptosis–immune crosstalk framework, in which Sertoli cell ferroptotic susceptibility, BTB disruption, DAMP–mediated macrophage activation, and adaptive immune responses are integrated as interconnected but experimentally testable events. This framework does not replace existing ferroptosis models but extends them by incorporating tissue–specific cellular interactions, particularly the vulnerability of Sertoli cells, disruption of the blood–testis barrier, and immune remodeling within the testicular microenvironment. Such a perspective may help explain how localized oxidative and metabolic disturbances evolve into persistent immune–mediated reproductive dysfunction.

First, a significant debate remains as to whether ferroptosis acts as a “primary initiator” or a “secondary amplifying event” in IR-induced testicular injury. Proponents of the “primary effect” argue that when IR acts on biological media, it instantaneously generates highly reactive hydroxyl radicals (•OH) via water radiolysis on a femtosecond-to-microsecond timescale. Given the high oxygen consumption and abundance of polyunsaturated fatty acids (PUFAs), particularly docosahexaenoic acid (DHA), in testicular tissue, this exogenous oxidative stress can directly abstract hydrogen atoms from membrane lipids of germ cells and Sertoli cells without relying on classical transcriptional networks, rapidly triggering a lipid peroxidation (LPO) cascade (28, 121). Such physicochemical direct membrane lipid damage can exceed the antioxidant buffering capacity of the endogenous GSH/GPX4 and FSP1 systems very early after irradiation, directly leading to membrane disintegration and ferroptosis (27). In contrast, proponents of the “secondary effect” point out that in the complex testicular microenvironment, large-scale ferroptosis is more likely a delayed pathological event secondary to DNA damage responses and subsequent microenvironmental disturbances (58). IR-induced DNA double-strand breaks persistently activate the ATM-p53 signaling axis, transcriptionally repressing SLC7A11 (the core subunit of System Xc^-^), leading to gradual GSH depletion and GPX4 inactivation (122). More importantly, DAMPs such as HMGB1 and mitochondrial DNA (mtDNA) released from early apoptotic germ cells activate resident macrophages to polarize toward a pro-inflammatory M1 phenotype (11). M1 macrophages secrete tumor necrosis factor-α (TNF-α), interleukin-1β (IL-1β), and nitric oxide (NO), which via paracrine effects disrupt iron metabolism and GPX4 stability in adjacent Sertoli cells, thereby inducing diffuse “bystander ferroptosis” (123, 124). This “primary vs. secondary” debate is not purely theoretical; it directly determines the critical time window for antioxidant versus anti-inflammatory interventions in radioprotection. Therefore, future studies urgently need to employ high-resolution real-time intravital imaging (e.g., C11-BODIPY live lipid peroxidation tracing) and single-cell spatial transcriptomics to precisely dissect and quantitatively characterize these two pathological mechanisms along the spatiotemporal axis.

Second, the central hub role of Sertoli cells in the testicular ferroptosis network may be significantly underestimated. Traditional reproductive toxicology has often viewed germ cells as the primary targets of radiation injury, but accumulating evidence indicates that the reversibility of testicular function depends critically on the structural and functional integrity of Sertoli cells and the BTB. Sertoli cells, as key maintainers of spermatogenic microenvironment stability, iron transport, and metabolic support, have high mitochondrial abundance and active iron metabolism, rendering them highly susceptible to iron-dependent lipid peroxidation under radiation stress (38). The pathological consequences of Sertoli cell ferroptosis are markedly amplified: reactive aldehyde products (e.g., 4-HNE) generated during lipid peroxidation can directly degrade or oxidatively modify tight junction proteins such as Claudin-11, Occludin, and ZO-1, driving the BTB from functional disturbance to anatomical barrier disruption (125). Once BTB physical integrity is lost, germ cell-specific antigens become exposed to the humoral immune system, triggering severe autoimmune reactions and chronic sterile inflammation. Thus, IR–induced male infertility may involve not only germ–cell loss but also disruption of testicular immune–microenvironment homeostasis, with Sertoli cell ferroptotic injury representing one potential contributing factor. This conceptual shift suggests that future targeted interventions should move from simply protecting germ cells to systematically maintaining the coordinated homeostasis of the “Sertoli cell–BTB–local immune niche”.

Third, current theoretical models of ferroptosis-immune crosstalk mostly adopt paradigms from tumor immunology or neurodegenerative diseases and fail to adequately consider the testis-specific immune privilege property (126). The testis is physiologically in a state of profound immune tolerance (18). Whether DAMPs released from ferroptosis are sufficient to completely remodel this immune-privileged microenvironment remains lacking direct *in vivo* molecular evidence. Although some studies have focused on classical inflammatory signaling pathways such as HMGB1/TLR4/NF-κB, the mechanisms of adaptive immune cell infiltration, the establishment of autoimmune memory, and the evolution of chronic sterile inflammation remain insufficiently elucidated. In particular, whether the testis exhibits low-grade immune activation similar to chronic systemic inflammation under low-dose, long-term chronic radiation exposure urgently requires the establishment of specific models for systematic investigation.

Fourth, the biological significance of ferroptosis in radiation injury needs to be evaluated dialectically. Although the mainstream view defines ferroptosis as a pathological damage mechanism to be inhibited, from the perspective of evolution and germ cell genetic integrity maintenance, moderate ferroptosis may serve a physiological clearance function. The testis is an organ dedicated to maintaining high genetic fidelity; rapid removal of abnormal spermatogonia or spermatocytes with severe DNA damage via ferroptosis could effectively prevent transmission of genetic mutations to offspring, suggesting that ferroptosis might have a reasonable “active compensatory clearance” role under certain pathophysiological conditions (127, 128). Indiscriminate, excessive inhibition of ferroptosis in clinical settings might lead to the abnormal survival of germ cells carrying potentially mutagenic risks, thereby increasing long-term genetic risks in offspring, for which adequate safety evaluations are still lacking. This “double-edged sword” nature warns that future therapeutic strategies should aim not at absolute blockade of ferroptosis pathways but at refined regulation of its pathological threshold and specific intervention windows (11).

Fifth, the non–linear dose–response relationship and temporal kinetics represent major challenges for understanding ferroptosis–associated radiation injury and its clinical translation. Most current studies investigating ferroptosis in radiation–induced testicular injury are based on acute high–dose exposure models, whereas occupational exposure and clinical radiotherapy more commonly involve low–dose, chronic, or fractionated irradiation (129). Importantly, low–dose radiation may not directly induce ferroptotic damage but instead activate adaptive antioxidant responses, including NRF2–mediated upregulation of SLC7A11 and other ferroptosis–resistance pathways (111, 112). However, persistent radiation exposure may gradually disrupt iron homeostasis and antioxidant capacity, allowing ferritinophagy–associated iron accumulation and lipid peroxidation to exceed cellular defense thresholds (14). Therefore, the biological outcome of radiation exposure likely depends on the dynamic balance between ferroptotic stress and adaptive protection, which determines whether cells undergo irreversible injury or maintain homeostasis. Furthermore, the translational application of ferroptosis–targeting interventions requires careful consideration of exposure patterns, intervention timing, BTB permeability, and long–term reproductive safety (130–132).

At the translational medicine level, although current ferroptosis-targeting intervention strategies show promise in animal experiments, they remain far from clinical application. Although small-molecule iron chelators, Ferrostatin-1/Liproxstatin-1, NRF2 agonists, and HMGB1 antagonists can alleviate acute oxidative stress and reduce BTB damage, once BTB anatomical disruption and autoimmune network activation occur, simply blocking lipid peroxidation cannot reverse the established pathological cascade (133). Therefore, future therapeutic regimens should evolve from single-target inhibition to multi-target, multi-stage combination strategies encompassing “precise iron homeostasis regulation–immune microenvironment remodeling –BTB structural repair–spermatogonial stem cell differentiation protection”.

In summary, current evidence supports considering ferroptosis as a potentially important component of IR–induced male reproductive injury, while the causal organization of ferroptotic and immune events remains incompletely defined. We therefore propose that conceptualizing these processes as an interconnected immune–metabolic network may provide a useful hypothesis–generating framework for future research. The validity of this framework will depend on direct spatiotemporal and cell–specific validation of its key links, particularly the proposed sequence connecting Sertoli cell ferroptosis, BTB dysfunction, immune activation, and secondary cellular injury. Human testicular organoids, spatial multi–omics, single–cell temporal analyses, and longitudinal clinical studies may help determine which components of the proposed network are causally relevant and therapeutically actionable.
